# Inhibition of Transglutaminase 2 Preserves Blood–Brain Barrier Integrity and Improves Neurological Outcomes After Experimental Traumatic Brain Injury in Mice

**DOI:** 10.1002/cns.70887

**Published:** 2026-04-19

**Authors:** Jieru Yang, Lihan Zhang, Xiancheng Qiu, Jiyang Wang, Ruicheng Yan, Jiasen Ye, Jianhua Peng, Yong Jiang

**Affiliations:** ^1^ Department of Neurosurgery, the Affiliated Hospital Southwest Medical University Luzhou China; ^2^ Laboratory of Neurological Diseases and Brain Function, the Affiliated Hospital Southwest Medical University Luzhou China; ^3^ Clinical Trial Research Center, the Affiliated Traditional Chinese Medicine Hospital Southwest Medical University Luzhou Sichuan China; ^4^ Academician (Expert) Workstation of Sichuan Province, the Affiliated Hospital, Southwest Medical University Luzhou China; ^5^ Institute of Brain Science Southwest Medical University Luzhou China; ^6^ Sichuan Clinical Research Center for Neurosurgery, the Affiliated Hospital Southwest Medical University Luzhou China

**Keywords:** blood–brain barrier, IL‐17, MMP‐9, transglutaminase 2, traumatic brain injury

## Abstract

**Background:**

Traumatic brain injury (TBI) is a leading global cause of disability and mortality, with blood–brain barrier (BBB) disruption exacerbating secondary injury. Transglutaminase 2 (TGM2), a multifunctional enzyme implicated in neuroinflammation and extracellular matrix remodeling, remains underexplored in TBI‐related BBB dysfunction. This study elucidates the role of TGM2 in BBB disruption following TBI and explores its therapeutic potential in mitigating BBB damage.

**Methods:**

The controlled cortical impact (CCI) and oxygen–glucose deprivation (OGD) models were used to establish in vivo and in vitro TBI models in mice. The experimental approaches comprised RNA sequencing, Western blot analysis, RT‐qPCR, immunofluorescence staining, and behavioral assessments.

**Results:**

TGM2 expression peaked at 48 h after TBI, predominantly in brain endothelial cells, correlating with BBB disruption (reduced tight junction proteins, increased edema). TGM2 knockdown may attenuate MMP‐9 via the IL‐17 pathway, restoring BBB integrity. In vivo, TGM2 inhibition reduced Evans blue leakage, upregulated CLAUDIN‐5/ZO‐1, and improved motor coordination, balance, and spatial memory.

**Conclusion:**

TGM2 is a key molecule affecting the BBB after TBI. Inhibition of TGM2 can alleviate the blood–brain barrier and neurological deficits after TBI, acting through the IL‐17‐MMP‐9 axis.

## Graphical abstract

The article unraveled that inhibition or knockdown of TGM2 can restore blood–brain barrier (BBB) permeability and ameliorate neurological deficits and cognitive dysfunction in a mouse model of traumatic brain injury (TBI). Mechanistically, TGM2 likely modulates the IL‐17 signaling pathway to regulate MMP9 expression, thereby affecting BBB permeability.

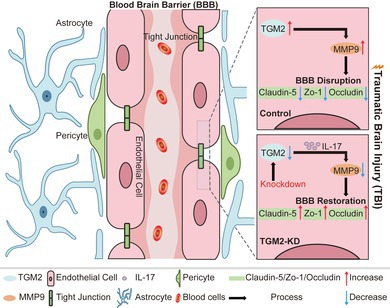

## 1 Introduction

Traumatic brain injury (TBI), a pervasive condition that spans across all age groups, stands as a primary contributor to mortality and disability globally [1]. TBI imposes a significant economic and psychological burden on patients and their families, while also posing a major challenge to public health systems. TBI pathophysiology is complex and unfolds through a biphasic process comprising primary and secondary injury mechanisms [1]. The primary injury occurs at the moment of trauma, involving direct mechanical disruption of brain tissue, blood vessels, and the blood–brain barrier (BBB) [2]. This initial insult triggers a cascade of biochemical and cellular events that drive secondary injury, which can persist from hours to days following the trauma. Secondary injury is characterized by a range of interconnected processes, including excitotoxicity, oxidative stress, BBB breakdown, neuroinflammation, mitochondrial dysfunction, and both apoptotic and necrotic cell death [3]. Among these processes, the disruption of BBB integrity plays a pivotal role, serving both as a marker and mediator of ongoing neural injury. Compromise of the BBB exacerbates cerebral edema, permits infiltration of peripheral immune cells, and amplifies inflammatory responses, thereby worsening neurological outcomes [4].

The BBB, a selectively permeable membrane, comprises a harmonious interplay of endothelial cells, the extracellular matrix components of the basement membrane, pericytes, and astrocyte terminals [5]. At the molecular level, BBB breakdown is primarily initiated by inflammatory cascades. Pro‐inflammatory cytokines such as tumor necrosis factor‐α (TNF‐α) and interleukin‐1β (IL‐1β) are rapidly upregulated following CNS injury, leading to downregulation and redistribution of key tight junction (TJ) proteins, including CLAUDIN‐5, OCCLUDIN, and ZO‐1 [6]. Concurrently, the activation of matrix metalloproteinases (MMPs), particularly MMP‐2 and MMP‐9, contributes to the proteolytic degradation of both extracellular matrix components and TJ‐associated proteins, further compromising BBB integrity [7]. In addition, infiltration of peripheral immune cells across the compromised BBB exacerbates neuroinflammation and promotes further tissue injury. This creates a vicious cycle in which barrier disruption and inflammation perpetuate each other, worsening neurological outcomes [6]. Endothelial cells are the main structural part of the blood–brain barrier (BBB). Under inflammatory and oxidative stress, these cells lose their barrier functions. Pericytes help control endothelial cell stability and blood vessel tone. During injury, pericytes separate from microvessels, weakening the BBB structure [8].

Tissue transglutaminase (TGM2), also known as type 2 transglutaminase, is a multifunctional enzyme with a molecular weight of 78 kDa, belonging to a multigene family [9]. Moreover, the role of TGM2 extends far beyond its enzymatic activity, as it plays a crucial role in cell growth, differentiation, apoptosis, inflammation, tissue repair, and fibrosis [10]. Furthermore, TGM2 is recognized as a key factor in neuroinflammation [11]. TGM2 has been implicated in the pathogenesis of various diseases beyond its physiological roles. In neurodegenerative disorders such as Alzheimer's disease, TGM2 promotes protein aggregation and neuronal apoptosis [12]. In cancers, TGM2 enhances tumor invasion, metastasis, and drug resistance by modulating extracellular matrix remodeling and activating survival signaling pathways [13]. Additionally, TGM2 contributes to tissue fibrosis by crosslinking extracellular matrix proteins and is recognized as a key autoantigen in celiac disease [14]. Despite its diverse functions, the specific mechanisms by which TGM2 regulates BBB disruption following TBI remain unclear. According to the pathological mechanism of TBI, TGM2 may be one of the key targets of BBB injury after TBI, providing a novel direction for intervention and treatment for TBI.

This study aims to investigate the role of TGM2 in regulating BBB integrity after TBI, and to elucidate the molecular mechanisms underlying this role. By combining animal models and cell experiments, we found that TGM2 may influence BBB integrity after TBI through the IL‐17‐MMP‐9 axis, suggesting the potential of TGM2 as a therapeutic target for reducing BBB damage after TBI.

## 2 Methods

### 2.1 Animals

Approval for all animal procedures described herein was granted by the China Committee for the Care and Use of Laboratory Animals and by the Institutional Animal Studies Committee of Southwest Medical University (reference no. 201903–105). Mice used in the experiments were provided by Chengdu Dashuo Experimental Animal Co. Ltd. The animals consisted of healthy male C57BL/6J mice aged 6–8 weeks, with an average body weight of 18–20 g. Housing of mice took place in the Medical Animal Center of the Affiliated Hospital of Southwest Medical University, where specific pathogen‐free (SPF) conditions were maintained through an Individually Ventilated Cage (IVC) system. The facility maintained optimal environmental parameters: Temperature at 24°C–25°C, relative humidity 55%–65%, and a 12‐h light/dark cycle (07:00–19:00 light phase) with minimized noise exposure during dark periods. All animals have free access to fresh feed and drinking water throughout the experimental period. The above recording of this research adheres to the ARRIVE 2.0 guidelines. Every in vivo experiment has been described in the figures' legends; for instance, it was determined by reference to previous studies on the CCI model and initial experimental results. Animals were randomly assigned to each group, and the observer remained unlabeled when conducting behavior tests and quantitative analyses (such as motor function screening, immunohistochemical, or Western blot experiments). Standard exclusion criteria involved dural injury during craniotomy or incapacity to recover from surgical sedation; excluded subjects were removed from subsequent statistics. Detailed animal usage is listed in Table S1.

### 2.2 Construction of CCI Injury Model

Anesthesia was induced in adult male C57BL/6 mice using pentobarbital sodium (60 mg/kg). The animals were then secured in a stereotaxic apparatus with body temperature maintained at 37°C. Following ethanol disinfection, a 10‐mm midline incision was made through the scalp to expose the cranium. A 4‐mm craniotomy was created in the right parietal bone, 1 mm lateral to the sagittal suture. Any mouse with evidence of dural perforation was excluded from further analysis. Traumatic brain injury (TBI) was induced using a controlled cortical impact (CCI) device equipped with a 3‐mm convex tip [15]. The impactor is perpendicular to the cortical surface, delivered parameters mimicking moderate TBI: 3 m/s velocity, 1.5 mm depth, and 100 ms dwell time [16]. Post‐impact bleeding was treated with sterile cotton, and the wound suture thread used was 6–0 silk. Mice recovered in heated cages before returning to housing. Sham‐operated controls underwent identical procedures, excluding CCI^17^.

### 2.3 Cell Culture and Oxygen–Glucose Deprivation (OGD) Model

Mice brain endothelial cell line Bend.3 (Cat# M‐C2007) was cultivated in Dulbecco's modified Eagle's medium (DMEM) (Cat# C11995500BT, Gibco) containing 10% fetal bovine serum (FBS) (Cat# 04–001‐IACS, Biological Industries) and 1% penicillin–streptomycin and maintained under a stable environment with 37°C and 5% CO2. When the cells were grown to 70%–80% in 6‐well plates, the medium containing serum was removed and replaced with glucose‐free medium. Under the condition of 37°C, 5% CO2, and 1% O2, cells were cultivated to simulate an environment causing cell injury in vivo [17].

### 2.4 TGM2‐KO Cell Line Construction and TGM2 Inhibitor Dosing Regimen in Mice

The TGM2‐targeting virus was acquired from OBiO Technology (Shanghai) Corp. Ltd., and the target sequence was 5′‐CCTGACAGAGTCAAACCTCAT‐3′. Add the virus to a 24‐well plate to infect Bend3 cells for 24 h. Subsequently, fresh medium was replaced, and puromycin (1 μg/mL) was added to screen for stably transfected cell lines. RT‐qPCR and WB were used to detect the knockdown efficiency. The TGM2 inhibitor Cystamine (purchased from MedChemExpress, MCE) was dissolved in phosphate‐buffered saline (PBS) at a concentration of 100 mg/mL according to the manufacturer's instructions. Mice were injected with Cystamine intraperitoneally at a dose of 150 mg/kg per day for 21 days, and then the mouse CCI model was established.

### 2.5 RNA Extraction and RT‐qPCR With Reverse Transcription

Tissues or cells were washed with PBS and then lysed directly by RNA isolator Total RNA Extraction Reagent (Cat# R401‐01, Vazyme). Cellular RNA was extracted using RNAeasy Isolation Reagent (Cat# R701‐02, Vazyme) according to the manufacturer's instructions. RT‐qPCR was carried out using ChamQ Universal SYBR qPCR Master Mix (Cat# Q711‐02/03, Vazyme) with the Real‐Time PCR Detection System. The relative expression of target genes was calculated using the 2 − ΔΔCt method, normalized to the housekeeping β‐actin. The results are expressed as a relative change from the control [18]. All primer sequences are listed in Table S2.

### 2.6 Western Blot (WB)

RIPA buffer was used to prepare tissue and cell lysates under ice‐incubation for 30 min, followed by centrifugation (12,000 × g, 10 min, 4°C). Protein‐containing supernatants were denatured at 100°C for 10 min in loading buffer. The samples were subjected to electrophoresis on a 10% SDS‐PAGE gel, transferred onto PVDF membranes, and blocked with 5% skim milk powder for 1 h. The samples were added to a 10% SDS‐PAGE gel for electrophoresis, after which the proteins were transferred to PVDF membrane, 5% skim milk powder was blocked for 1 h, and then overnight at 4°C with the following primary antibody: TGM2 (D11A6) XPRabbit mAb# 3557 (1:1000 Cell Signaling Technology Cat# 335 T), Claudin‐5 (E8F3D) Rabbit mAb# 49,564 (1:1000 Cell Signaling Technology Cat# 49564S); Beta Actin Monoclonal Antibody (1:4000 Proteintech Cat# 66009–1‐Ig), ZO‐1 Polyclonal antibody (1:1000 Proteintech Cat No. 21773–1‐AP), and Anti‐MMP‐9 antibody (1:1000 Abcam ab38898). Then incubated with the following appropriate secondary antibodies (1:5000 Proteintech Cat# SA00001‐2) for 1 h at room temperature; the membrane was then washed with TBST for 10 min, three times. Signal intensity was imaged using a FDbio‐Dura ECL KIT (Cat# FD8020, Fdbio science) and analyzed with ImageJ software (NIH) [18].

### 2.7 Evans Blue (EB) Extravasation

Evans blue dye (0.5%) was intravenously administered via the retro‐orbital vein and allowed to circulate for 2 h. Mice were then deeply anesthetized with 1% sodium pentobarbital, and brain tissues were harvested. After fixation in 4% paraformaldehyde for 30 min, tissues were embedded in OCT compound and immediately frozen. Coronal sections (40 μm thickness) were prepared using a cryostat. Sections were incubated with 10 μL of DAPI staining solution and mounted. Evans blue extravasation around lesion sites was visualized and imaged using a confocal microscope.

For quantification, Evans blue leakage was assessed from confocal images using ImageJ by an investigator blinded to group assignment. For each mouse, 6 coronal sections spanning the perilesional cortex were analyzed. Evans blue‐positive area (or mean fluorescence value after background subtraction) in the specified Region of Interest was obtained, and their integrals were summed up to form specific parameters for each group, subsequently analyzed statistically.

### 2.8 Brain Water Content

Using the wet‐dry method to determine brain water content. The entire brain was harvested at 48 h after TBI. The brain specimens were immediately weighed after harvest to obtain the wet weight and then dried at 105°C for 72 h to determine the dry weight. The percentage of brain water content was calculated as [(wet weight − dry weight)/wet weight] × 100% [19].

### 2.9 Immunofluorescence Staining

At 48 h following TBI, cardiac perfusion was performed using pre‐chilled PBS and 4% paraformaldehyde. Brains were removed, immersed in the same fixative for 30 min, and then dehydrated in 30% sucrose overnight at 4°C before OCT embedding. Tissue sections (10 μm) were subsequently subjected to antigen retrieval with 0.01 M sodium citrate, permeabilized with 0.3% Triton X‐100 for 10 min, and blocked with 10% goat serum for 1 h. TGM2 (D11A6) XPRabbit mAb# 3557 (1:100 Cell Signaling Technology Cat# 335 T), CLAUDIN‐5 (E8F3D) Rabbit mAb# 49,564 (1:100 Cell Signaling Technology Cat# 49564S), and ZO‐1 Polyclonal antibody (1:100 Proteintech Cat No. 21773–1‐AP) were kept overnight at 4°C, fluorescent secondary antibody Goat anti‐Rabbit (H + L) (1:200 Abcam Cat# ab150077, RRID: AB_2630356) and Goat anti‐Mouse (H + L) (1:200 Abcam Cat# ab150116, RRID: AB_2650601) were incubated for 1 h at room temperature (24°C–28°C), DAPI blocked, and observe the co‐localization of TGM2/CLADIN‐5/THBS1/LECTIN under a fluorescence microscope (Olympus Japan) [20]. The TBI group and the Sham group were photographed in the same place on the corresponding brain slices [21].

### 2.10 RNA‐Seq

Bend.3 were inoculated in a 6‐well plate, and after 12 h of complete cell wall adhesion, hypoxia and glucose deprivation environments stimulated the cells and reached the scheduled stimulation time. Cellular RNA was extracted using RNAeasy Isolation Reagent (#R701‐02, Vazyme Biotech) according to the manufacturer's instructions. The Novogene sequence was sequenced on an Illumina HiSeq 6000 high‐throughput sequencing system.

### 2.11 Transcriptome Analysis

The raw FASTQ files underwent adapter trimming and low‐quality read removal using Trim Galore (version 1.18). Quality control was subsequently performed with FastQC (version 0.11.9). The remaining reads were aligned to the GRCm38 mouse genome using HISAT2 (v2.2.0) with default parameters and filtered with samtools (version 1.10, parameters used: Samtools view‐F 1804‐f 2‐q 30). Gene‐level read counts were obtained with featureCounts (version 2.0.1) based on the Ensembl annotation (mm10). Subsequently, TPM (Transcripts Per Kilobase of exon model per Million mapped reads) in each gene was calculated for subsequent analysis. Differentially expressed genes (DEGs) were evaluated using the DESeq2 package in R (version 4.2.0), using adjusted *p* < 0.05 and |log_2_FC| > 2 as cutoffs to define the DEGs. Gene Ontology (GO) and Kyoto Encyclopedia of Genes and Genomes (KEGG) enrichment analyses were performed on the DEGs using the clusterProfiler package (version 4.0.5) in Bioconductor. Pathways with a false discovery rate (FDR) below 0.05 were considered significantly enriched [22].

### 2.12 Behavioral Tests

Modified Neurologic Severity Score (mNSS) was used to evaluate the degree of neurological deficit after traumatic brain injury [23]. Using a wire‐hanging test to determine an animal's muscular strength, as well as its coordination and endurance [24]. The balance beam test was used to evaluate the fine motor coordination and balance ability of the experimental animals [25]. The rotary‐rod test was used to evaluate the motor coordination and balance ability of small animals [26]. An open field test was used to evaluate the autonomic activity and exploratory behavior of mice [27]. The Morris water maze was used to test the learning ability and memory function of the mice [28]. All behavioral experimental procedures in this study were conducted according to the standard Procedure.

### 2.13 Y‐Maze Test

Spontaneous alternation behavior to assess spatial memory within a short time. Mice were allowed to explore the three identical arms of a Y‐shaped maze freely for 8 min. The order in which the arms were entered was recorded, and the percentage of spontaneous alternation was calculated as the number of triads containing entries into all three arms divided by the total number of possible alternations.

### 2.14 Novel Object Recognition (NOR) Test

Mice were habituated to an empty open field arena (40 cm × 40 cm) for 24 h. And two identical objects were set up in the arena for a 5‐minute familiarization session. After a 4 h interval, the familiar object was substituted with a novel one, followed by an 8‐min exploration period. Exploration durations for both objects were recorded. The recognition index was calculated by dividing the time spent with the novel object by the total exploration time and multiplying by 100%.

### 2.15 Statistical Analysis

GraphPad Prism 9.5 was used for statistical analysis. Using the Shapiro–Wilk normality test to assess normality. We used an unpaired two‐tailed *t*‐test to compare two groups. One‐way ANOVA with Fisher's LSD post hoc test was used to perform multiple comparisons among group means. Bar graphs were described as mean ± Standard error of the mean (SEM) of at least three independent experiments. For non‐normally distributed data, we employed the Mann–Whitney test for two‐group comparisons and the Kruskal–Wallis test for multiple groups. *p* < 0.05 was considered statistically significant and is denoted as follows: *p < 0.05; **p < 0.01; ***P < 0.001 [18].

### 2.16 Histological and Immunohistochemical Analysis

Serial coronal brain sections from the Sham, TBI‐24 h, TBI‐48 h, TBI‐48 h + Cystamine, TBI‐72 h, and TBI‐30 days post‐TBI groups (*n* = 3/group) were stained with Hematoxylin and Eosin (H&E) for lesion volume quantification. The lesion percentage was calculated using ImageJ software by an investigator blinded to the experimental groups.

Immunohistochemical (IHC) analysis of brain sections was performed using primary antibodies against Anti‐SUR1 antibody (1:200, Abcam, ab217633), TRPM4 Polyclonal antibody (1:200, Proteintech, 21,985–1‐AP), Anti‐NKCC1 antibody (1:200, Abcam, ab303518), and Aquaporin 4 Polyclonal antibody (1:200, Proteintech, 16,473–1‐AP). After incubation with appropriate secondary antibodies, the images were captured using a light microscope at 4X and 20X magnification. Using the ImageJ software for quantitative analysis of staining intensity.

## 3 Results

### 3.1 TGM2 Might Be One of the Key Proteins Involved in the Breakdown of the BBB After TBI

In our earlier studies [22], high‐throughput RNA sequencing and proteomic sequencing were performed on Sham and injured hemispheres of the TBI model. A total of 37,908 genes and 3621 proteins were quantified. We identified 581, 208, and 868 DEGs at 12 h, 24 h, and 72 h, respectively. GO enrichment analysis showed that the pathological mechanisms of blood–brain barrier disruption include the upregulation of pathways such as reactive oxygen species metabolic process, regulation of tumor necrosis factor (TNF) production, and regulation of angiogenesis throughout 12 h, 24 h, and 72 h after TBI (Figure S1A). We conducted WGCNA analysis on the 3621 proteins that were identified. The result showed that the yellow module, which had a strongly negative correlation with the Sham group, was −0.91 (Figure S1B). By intersecting the 137 proteins in the yellow module with the differentially expressed genes at three time points, we found that TGM2 might be one of the key proteins associated with the disruption of the blood–brain barrier after TBI (Figure S1C).

**FIGURE 1:**
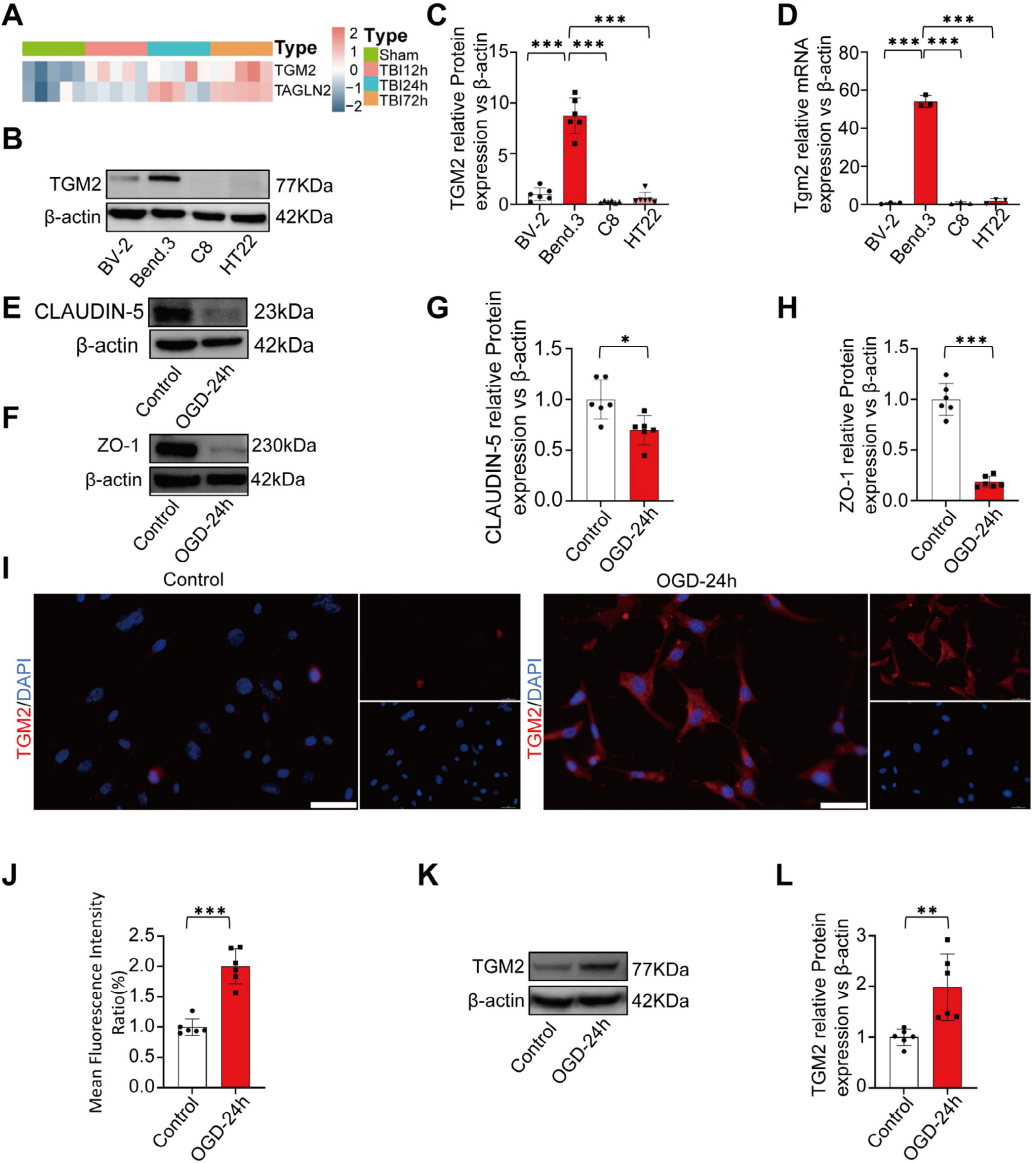
The expression of TGM2 and disruption of BBB integrity in Bend.3 after OGD. (A) Heatmap of *Tgm2* and *Tagln2* mRNA expression after TBI at 12 h, 24 h, and 72 h vs. Sham groups. (B, C) Protein quantification in four brain cell lines, *n* = 6 (independent cell culture preparations) in each group. (D) *Tgm2* mRNA levels across four cell lines, *n* = 3 (independent cell culture preparations) in each group. (E–H) CLAUDIN‐5/ZO‐1 protein expression in OGD‐24 h vs. the control group. (I, J) Immunofluorescence staining of TGM2 after OGD at 24 h, Scale bar = 50 μm. (K, L) TGM2 protein quantification in OGD‐24 h vs. the control group, *n* = 6 (independent cell culture preparations) in each group. All data are shown as the mean ± SEM. *p < 0.05, **p < 0.01, ***p < 0.001 vs. Control/OGD group. BV‐2: Mouse microglial cell line. Bend.3: Mouse brain microvascular endothelial cell line. C8: Mouse cerebellar astrocyte cell line. HT22: Mouse hippocampal neuronal cell lines. OGD: Oxygen Glucose Deprivation. TBI, Traumatic brain injury.

Transcriptomic analysis of mouse brain tissue revealed an increase in *Tgm2* expression after TBI at 12 h, 24 h, and 72 h (Figure 1A). To determine which type of brain cells exhibited the highest expression of TGM2, we performed WB and RT‐qPCR analysis on mouse microglia, astrocytes, brain endothelial cells, and neurons. The results showed that TGM2 expression was highest in mouse brain endothelial cells (Figure 1B–D). Subsequently, we modeled OGD in mouse brain endothelial cells. WB analysis of BBB integrity markers revealed a significant decrease in the expression of CLAUDIN‐5 and ZO‐1 after OGD (Figure 1E–H), indicating BBB integrity was impaired. Finally, immunofluorescence staining (Figure 1I,J) and WB (Figure 1K,L) revealed that TGM2 expression was increased in Bend.3 cells after OGD.

### 3.2 Increased Expression of TGM2 and Disruption of BBB Integrity Following TBI

**FIGURE 2:**
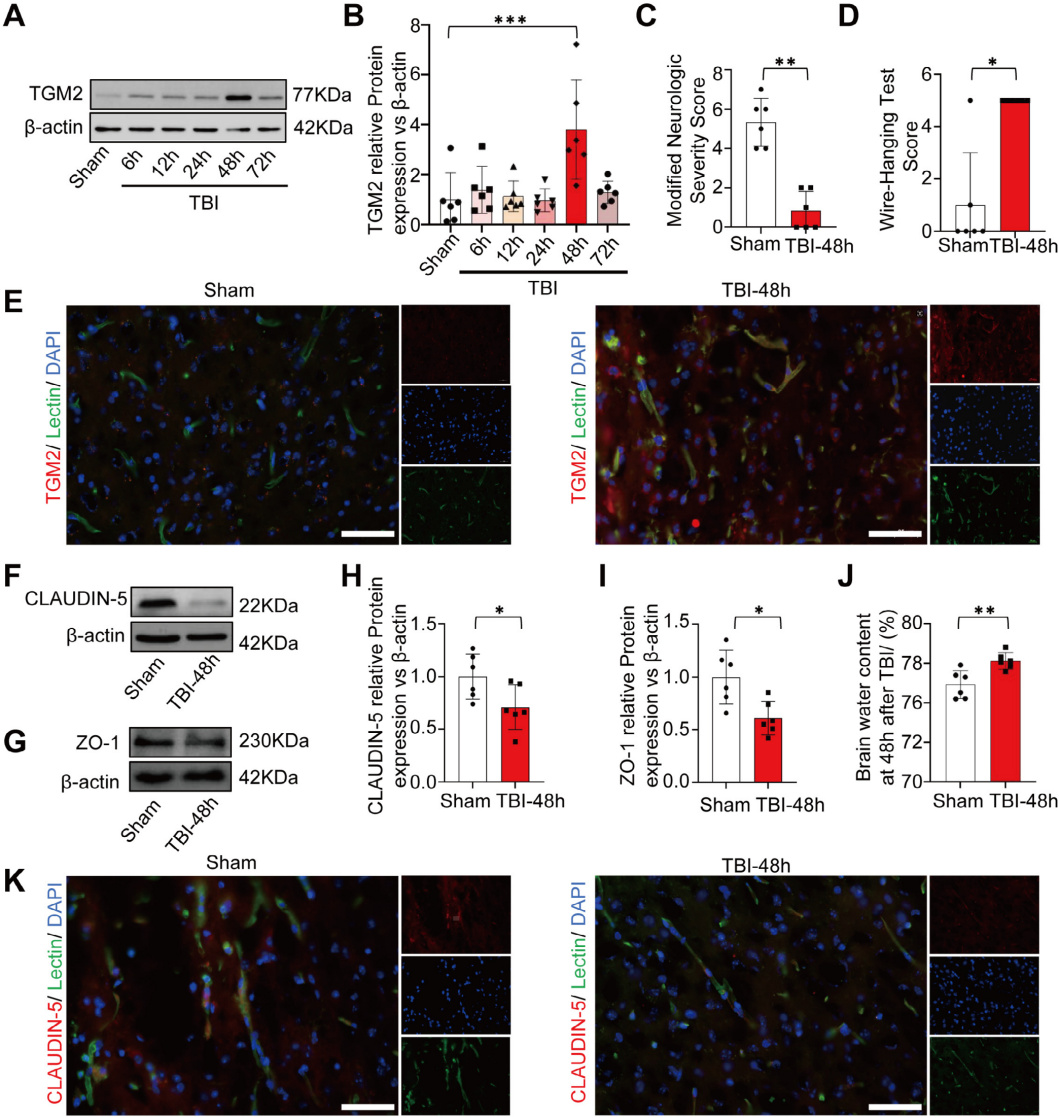
The expression of TGM2 and disruption of BBB integrity after TBI. (A, B) Time course of TGM2 expression in the brain after TBI, *n* = 6 mice/group. (C, D) Neurological deficits assessed by mNSS and wire‐hanging tests, *n* = 6 mice/group. (E) Dual immunofluorescence of TGM2(red) and lectin(green) for co‐localization, Scale bar = 25 μm. (F–I) CLAUDIN‐5 and ZO‐1 protein quantification after TBI, *n* = 6 mice/group. (J) Brain water content quantification, *n* = 6 mice/group. (K) Immunofluorescence shows CLAUDIN‐5 and lectin co‐staining. Scale bar = 25 μm. All data are shown as the mean ± SEM. *p < 0.05, **p < 0.01, ***p < 0.001 vs. Sham/TBI‐48 h group. TBI: Traumatic brain injury; mNSS: Modified neurologic severity score.

To identify the specific moment when TGM2 expression peaks after TBI in mice, we established experimental groups at Sham, 6 h, 12 h, 24 h, 48 h, and 72 h after TBI. WB analyses revealed that TGM2 expression was most significantly elevated at 48 h (Figure 2A,B), prompting the selection of this time point for subsequent animal experiments. Neurobehavioral assessments conducted at 48 h after TBI demonstrated a significant increase in mNSS and a decline in wire hang test results (Figures 2C,D), suggesting severe cognitive dysfunction. To confirm TGM2 expression in brain endothelial cells, co‐staining of TGM2 with lectin (a vascular marker) on brain sections showed TGM2 localization around blood vessels, with fluorescence intensity markedly enhanced in the perivascular regions after TBI (Figure 2E). WB analysis of BBB integrity markers showed significant downregulation of CLAUDIN‐5 and ZO‐1 at 48 h after TBI (Figure 2F–I). Additionally, brain water content measurement confirmed an increase at 48 h after TBI (Figure 2J), indicative of BBB disruption. Finally, immunofluorescence co‐staining of CLAUDIN‐5 and lectin demonstrated reduced CLAUDIN‐5 fluorescence intensity around blood vessels in TBI mice (Figure 2K), additionally supporting the conclusion that BBB integrity was impaired.

### 3.3 Effects of TGM2 Knockdown on BBB Integrity in Bend.3 Cells After OGD

**FIGURE 3:**
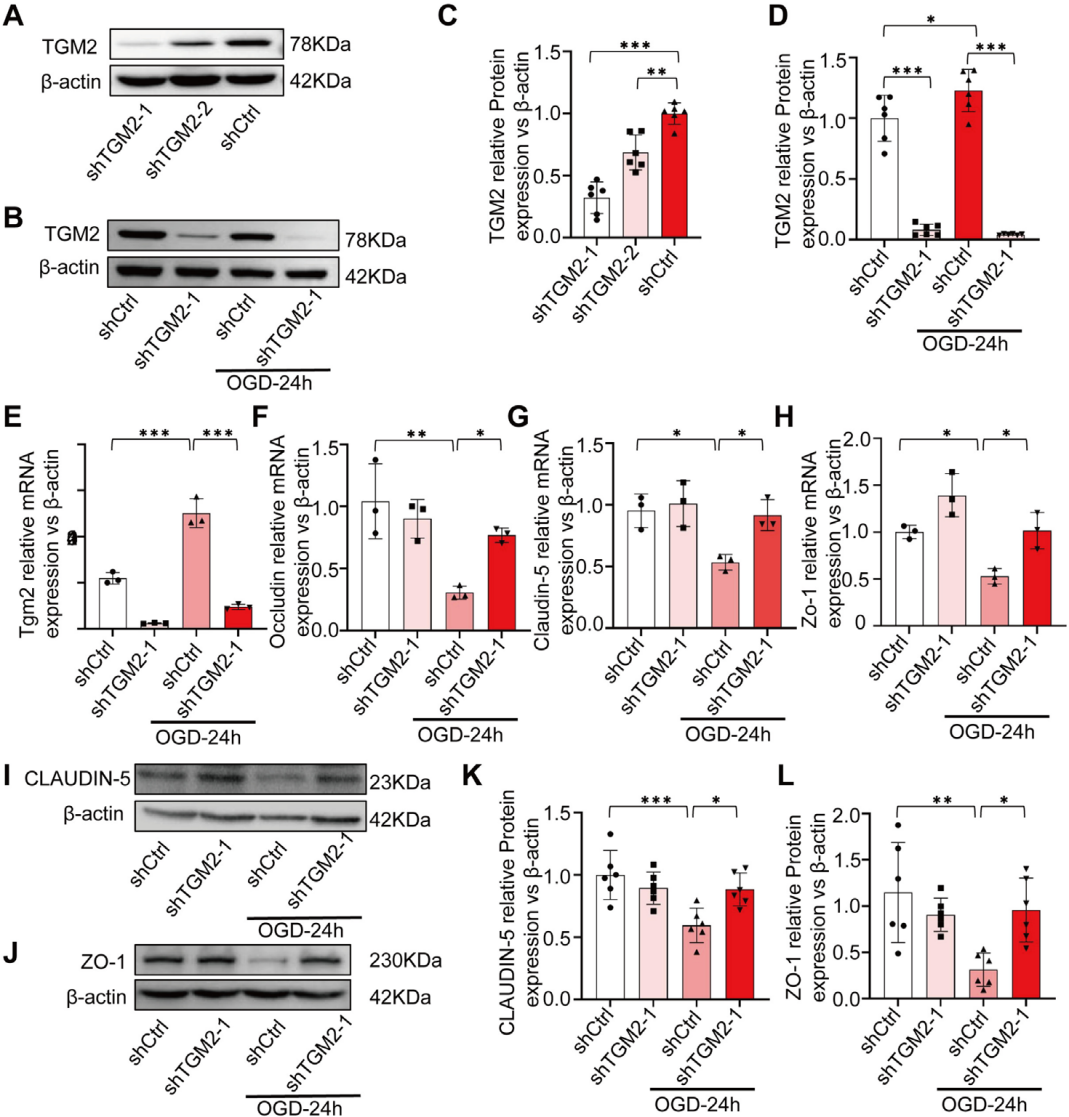
TGM2‐KD can regulate BBB relative protein expression in ECs. (A, C) TGM2 knocked down efficiency in the Bend.3, *n* = 6 (independent cell culture preparations) in each group. (B, D) OGD‐induced TGM2 protein alterations in shCtrl vs. shTGM2, *n* = 6 (independent cell culture preparations) in each group. (E–H) mRNA expression levels of *Tgm2*/*Occludin*/*Claudin‐5*/*Zo‐1*, *n* = 3 (independent cell culture preparations) in each group. (I–L) CLAUDIN‐5/ZO‐1 protein rescued by shTGM2 post‐OGD, *n* = 6 (independent cell culture preparations) in each group. All data are shown as the mean ± SEM. *p < 0.05, **p < 0.01, ***p < 0.001 vs. shCtrl/shTGM2 OGD24h +/− group. KD: Knockdown; BBB: Blood–brain barrier; ECs: Endothelial cells; OGD: Oxygen Glucose Deprivation.

To examine the influence of TGM2 knockdown on BBB integrity in Bend.3 cells after OGD, we first validated the efficiency of *Tgm2*‐specific lentiviral shRNA constructs by infecting cells with TGM2‐targeting lentiviruses. WB analyses showed that shTGM2‐1 was an effective knockdown sequence (Figure 3A,C). Cells were then subjected to OGD modeling and divided into four groups: shCtrl, shTGM2, shCtrl‐OGD, and shTGM2‐OGD. WB and RT‐qPCR analysis demonstrated that the shTGM2‐1 sequence effectively knocked down TGM2 even after OGD and confirmed that TGM2 expression was increased following OGD treatment alone (Figure 3B,D,E). Subsequent RT‐qPCR assessment of BBB integrity markers showed that *occludin*, *Claudin‐5*, and *Zo‐1* expression levels were lower in the shCtrl‐OGD group than in the non‐OGD group. In contrast, the shTGM2‐OGD group exhibited partial restoration of these markers, with expression levels higher than those in the shCtrl‐OGD group but lower than those in the shCtrl and shTGM2 groups (Figure 3F–H). These findings suggest that TGM2 knockdown mitigates OGD‐induced impairment of BBB integrity. WB analysis further supported these results. CLAUDIN‐5 and ZO‐1 protein expression in the shTGM2‐OGD group were higher than in the shCtrl‐OGD group, though still lower than in the shCtrl and shTGM2 groups (Figure 3I–L). Collectively, these findings demonstrate that TGM2 depletion partially revives BBB stability following OGD, emphasizing TGM2's possible function in modulating BBB stability under OGD environments.

### 3.4 TGM2 Knockdown Modulates MMP‐9 Expression via the IL‐17 Pathway to Preserve BBB Integrity in Bend.3 Cells After OGD

**FIGURE 4:**
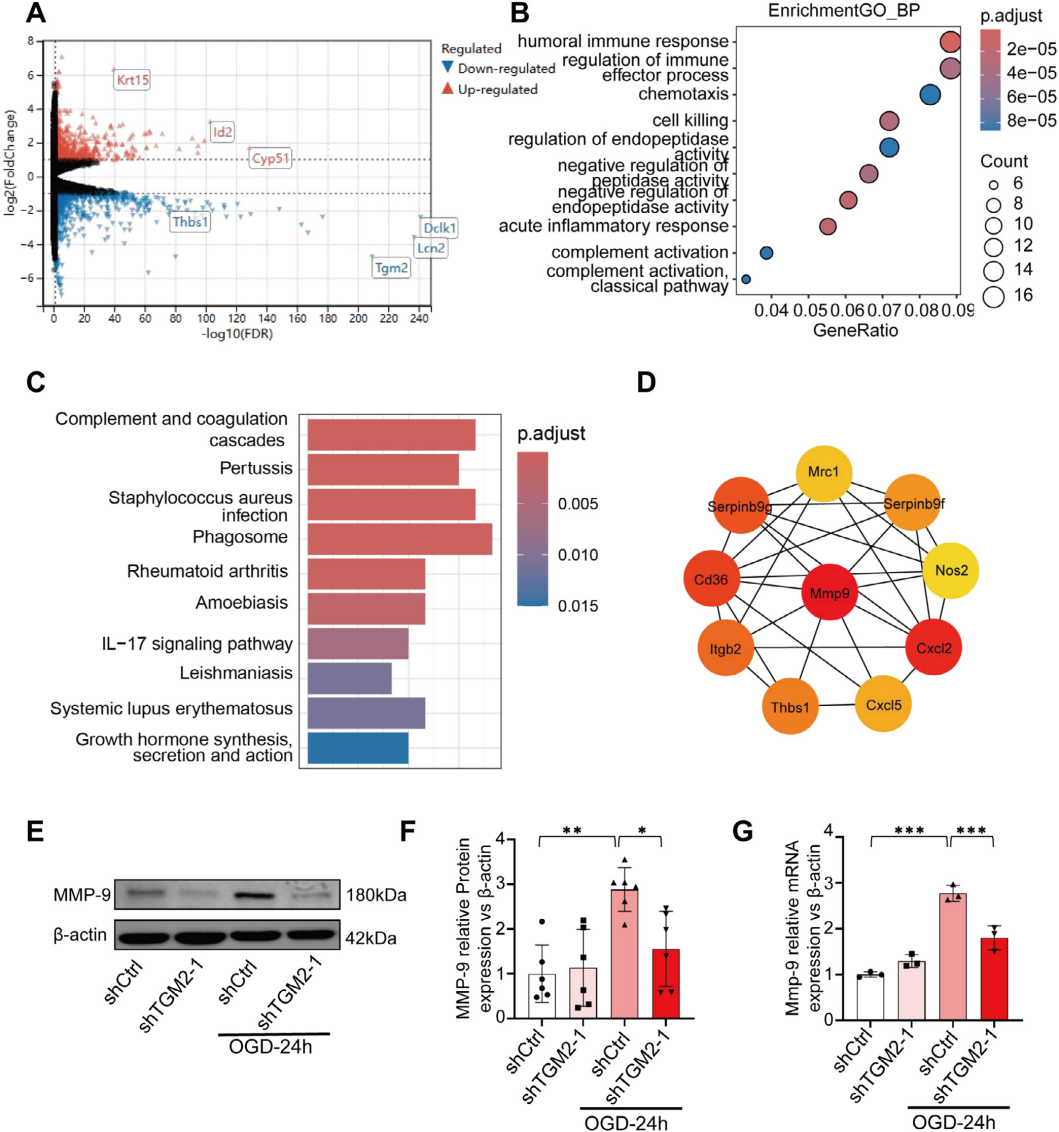
TGM2‐KD down‐regulates MMP9 expression through the IL‐17 pathway in Bend.3 after OGD. (A) Volcano plot of DEGs in TGM2‐KD vs. NC‐OGD group, with red points representing up‐regulated genes and blue points representing down‐regulated genes. (B, C) GO and KEGG enrichment analysis of down‐regulated DEGs. (D) The top 10 hub genes calculated by cytoHubba. (E, F) MMP9 protein suppression by shTGM2 post‐OGD, *n* = 6 (independent cell culture preparations) in each group. (G) mRNA expression level of Mmp9 in shTGM2 after OGD, *n* = 3 (independent cell culture preparations) in each group. All data are shown as the mean ± SEM. *p < 0.05, **p < 0.01, ***p < 0.001 vs. shCtrl+shTGM2 OGD24h +/− group. KD: Knockdown; OGD: Oxygen Glucose Deprivation; DEG: Differentially expressed genes. GO: Gene Ontology; KEGG: Kyoto Encyclopedia of Genes and Genomes; PPI: Protein–Protein Interaction.

In an OGD‐treated cell model, after knocking down TGM2 via lentiviral shRNA, we conducted high‐throughput sequencing. RNA sequencing revealed significant differential gene expression (shTGM2‐1 vs. shCtrl post‐OGD), with marked downregulation of *TGM2* (Figure 4A), confirming TGM2 knockdown was effective. Interestingly, it was observed that the mRNA levels of matrix metalloproteinase 9 (*Mmp‐9*) were significantly downregulated, with a log2(fold change) of −2.84, suggesting that knockdown of TGM2 might be involved in regulating the expression of *Mmp‐9*. GO analysis identified enriched biological processes, including humoral immunity, chemotaxis, and acute inflammatory responses (Figure 4B). Through KEGG enrichment analysis, we found that the downregulated DEGs were mainly enriched in pathways such as complement/coagulation cascades, IL‐17 signaling, and autoimmune pathways (Figure 4C). The genes in the top 10 enriched KEGG pathways were input into the STRING database to construct a protein–protein interaction network, and the EPC algorithm of CytoHubba was used to calculate hub scores [29]. The hub genes were obtained, and *Mmp‐9* was the top‐ranked gene (Figure 4D). In the top 10 KEGG results, *Mmp‐9* was involved only in the enriched IL‐17 signaling pathway, which demonstrates that Mmp‐9 serves as a critical gene possessing both biological significance and topological robustness within this network. WB confirmed reduced MMP‐9 protein levels in shTGM2 groups post‐OGD (Figure 4E,F). RT‐qPCR validated decreased *Mmp‐9* mRNA (Figure 4G), demonstrating that TGM2 knockdown suppresses the expression of MMP‐9. These findings suggest TGM2 inhibition may attenuate BBB dysfunction in TBI via regulating MMP‐9 through IL‐17 signaling.

### 3.5 In Vivo Inhibition of TGM2 Attenuates TBI‐Induced BBB Integrity Impairment

**FIGURE 5:**
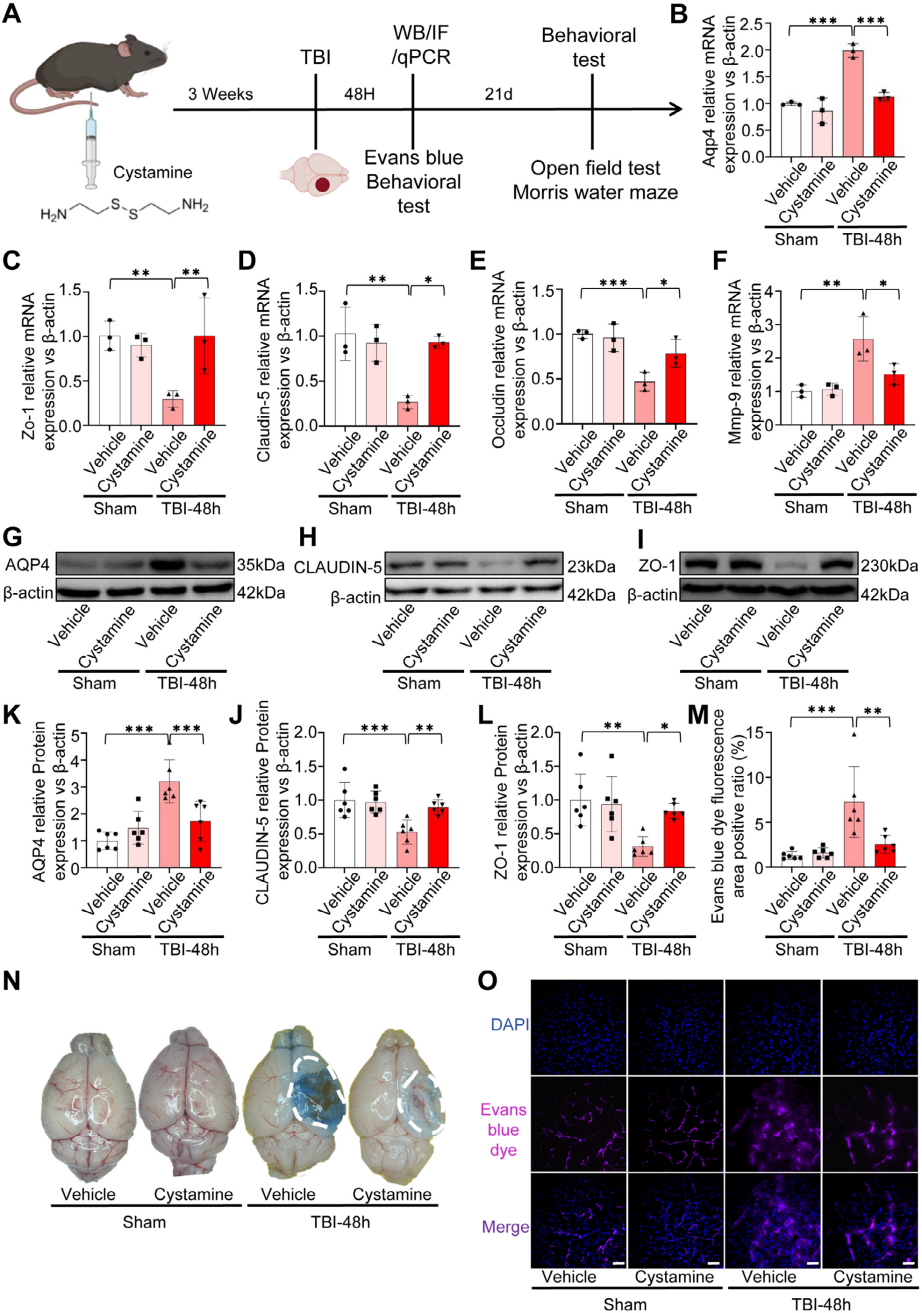
Effect of Cystamine (TGM2 inhibitor) on the BBB after TBI in mice. (A) Schematic of drug administration in mice. (B–F) mRNA expression of *AQP4*/*Occludin*/*Claudin‐5*/*Zo‐1*/*Mmp9*, *n* = 3 mice/group. (G–I) Representative Western blot imaging of AQP4、CLAUDIN‐5, and ZO‐1. (K–L) Protein quantification shows cystamine‐mediated AQP4 suppression and CLAUDIN‐5/ZO‐1 restoration, *n* = 6 mice/group. (M) Quantification of Evans blue extravasation, *n* = 6 mice/group. (N, O) Evans blue extravasation imaging, Scale bar = 20 μm. All data are shown as the mean ± SEM. *p < 0.05, **p < 0.01, ***p < 0.001 vs. Sham and vehicle/cystamine group. BBB: Blood–brain barrier; TBI: Traumatic brain injury.

To evaluate the effect of TGM2 inhibition on BBB integrity after TBI, we administered the TGM2 inhibitor cystamine in vivo. The experimental design and dosing regimen are illustrated in Figure 5A. To assess the impact of TGM2 inhibition on BBB integrity, we compared four groups: Sham+Vehicle, Sham+Cystamine, TBI + Vehicle, and TBI + Cystamine. RT‐qPCR results revealed that the expression levels of Aqp4 and Mmp‐9 were increased, whereas the expression levels of Zo‐1, Claudin‐5, and Occludin were decreased in the TBI + Vehicle group. In contrast, in the TBI + Cystamine group, Aqp4 and Mmp‐9 expression levels were reduced, while Zo‐1, Claudin‐5, and Occludin expression levels were elevated, approaching those observed in the Sham+Vehicle group (Figure 5B–F). WB results further showed that compared with the Sham+Vehicle group, the TBI + Vehicle group exhibited increased AQP4 expression and decreased CLAUDIN‐5 and ZO‐1 expression. Conversely, in the TBI + Cystamine group, AQP4 expression was reduced, whereas CLAUDIN‐5 and ZO‐1 expression levels were recovered (Figure 5G–L).

To directly assess BBB permeability, Evans blue dye extravasation assays demonstrated prominent dye leakage in the TBI + Vehicle group, while cystamine markedly attenuated Evans blue leakage after TBI (Figure 5N,O). Quantification of Evans blue fluorescence‐positive area ratio further confirmed a significant reduction in dye extravasation in the TBI + Cystamine group compared with the TBI + Vehicle group (Figure 5M). Collectively, these results indicate that pharmacological inhibition of TGM2 by cystamine mitigates BBB disruption and brain edema after TBI, accompanied by suppression of MMP‐9 upregulation.

### 3.6 TGM2 Inhibition Reduces Lesion Volume and Modulates the Expression of Edema‐Related Proteins

**FIGURE 6:**
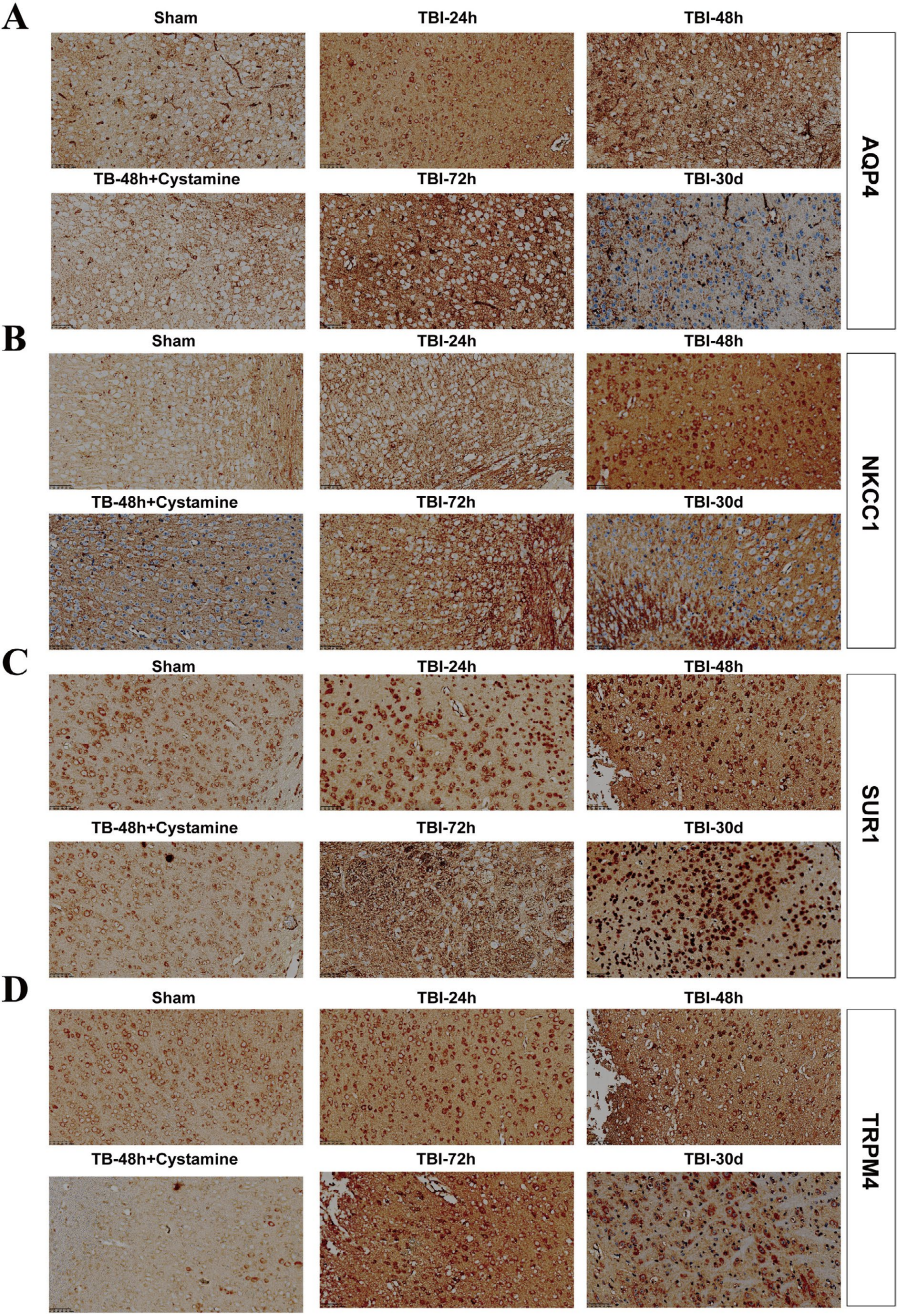
Representative immunohistochemical image of expression of brain edema‐related protein AQP4, NKCC1, SUR1, TRPM4 in the Sham group, the TBI‐24 h group, the TBI‐48 h group, the TBI‐48 h plus cysteamine group, the TBI‐72 h group, and the TBI‐30‐day group.

To further evaluate the neuroprotective effect of TGM2 inhibition, we assessed lesion volume via H&E staining at multiple time points post‐TBI. Quantitative analysis revealed that cystamine treatment significantly reduced the lesion percentage compared to the TBI + Vehicle group at 48 h (Figure S2). Furthermore, immunohistochemical analysis demonstrated an upregulation of SUR1, TRPM4, NKCC1, and AQP4 proteins in the ipsilateral cortex following TBI, which was substantially attenuated by cystamine treatment at 24 h, 48 h, 72 h, and 30 days post‐injury (Figure 6 and Figure S3). Consistent with these findings, RT‐qPCR analysis confirmed that the mRNA expression levels of *Aqp4*, *Nkcc1*, *Sur1*, and *Trpm4* were significantly increased after TBI, and this upregulation was suppressed by cystamine administration (Figure S4A). These results collectively indicate that pharmacological inhibition of TGM2 effectively mitigates secondary brain damage and edema formation following TBI.

### 3.7 TGM2 Inhibitor Cystamine Exerts Neuroprotective Effects in TBI Mice

**FIGURE 7:**
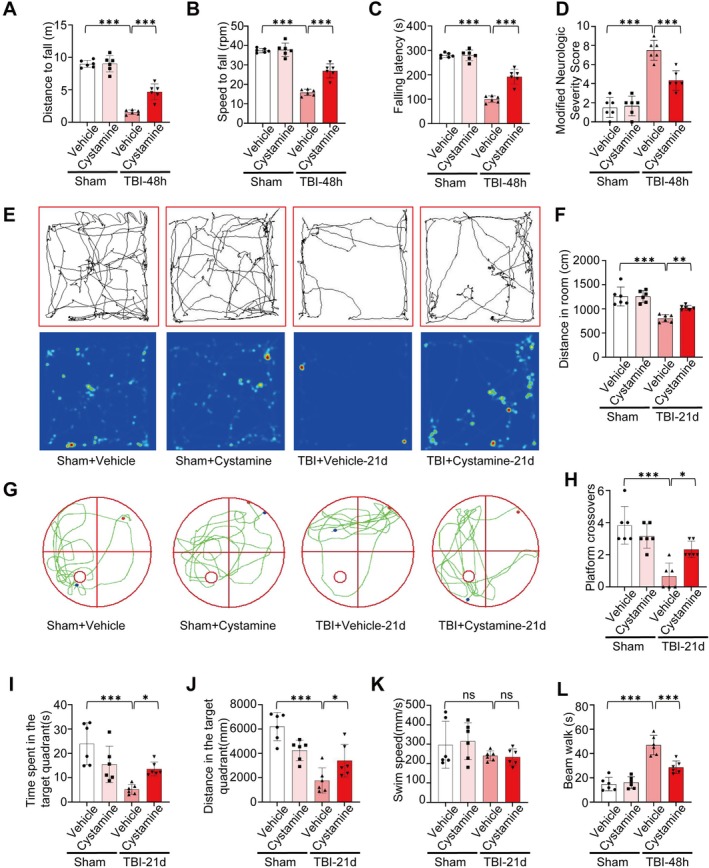
Effect of Cystamine (TGM2 inhibitor) on neuromotor function after TBI in mice. (A–C) Rotarod latency, *n* = 6 mice/group. (D) mNSS score, *n* = 6 mice/group. (E, F) Open field test (Trajectory diagram; trajectory heatmap; total distance), *n* = 6 mice/group. (G) Morris water maze trajectory diagram. (H–K) Morris water maze statistics of platform crossings, the time spent in the target quadrant, distance in the target quadrant, and swim speed, *n* = 6 mice/group. (L) Beam‐walk scores, *n* = 6 mice/group. All data are shown as the mean ± SEM. *p < 0.05, **p < 0.01, ***p < 0.001 vs. Sham and vehicle/cystamine group. mNSS: Modified neurologic severity score.

To assess the neuroprotective impact of the TGM2 inhibitor cystamine, a series of behavioral tests were conducted in mice. The rotarod test showed that TBI + Vehicle mice exhibited impaired motor coordination compared with Sham + Vehicle mice, whereas performance was partially restored in the TBI + Cystamine group (Figure 7A–C). mNSS assessment confirmed severe neurological deficits in the TBI + Vehicle group compared with the Sham group, which were significantly attenuated in the TBI + Cystamine group (Figure 7D). Additionally, the balance beam test revealed prolonged crossing times in the TBI + Vehicle group, which were significantly shortened following cystamine treatment (Figure 7L). Open field analysis demonstrated that exploratory movement was markedly reduced in TBI + Vehicle mice, while the TBI + Cystamine group exhibited partial recovery of exploratory behavior (Figure 7E,F). In the Morris water maze test, TBI + Vehicle mice displayed spatial memory impairments, including disordered search paths, reduced number of platform crossings, shortened target quadrant occupancy time, and decreased movement distance in the target quadrant, although swimming speed was similar across groups. Notably, target quadrant occupancy and crossing frequency were notably elevated in the TBI + Cystamine group (Figure 7G–K). And TBI mice displayed a longer escape latency compared to Sham mice, suggesting impaired spatial learning. Cystamine treatment significantly shortened the escape latency, demonstrating improved learning ability (Figure S5E). Meanwhile, we conducted the Y‐maze test and the novel object recognition test. In the Y‐maze test, TBI mice exhibited a significant reduction in the spontaneous alternation percentage, indicative of impaired working memory. This deficit was markedly improved by cystamine treatment (Figure S5C). The NOR test revealed that TBI mice spent significantly less time exploring the novel object, showing a decreased recognition index. Cystamine treatment restored the recognition index to a level comparable to the Sham group (Figure S6A,C). These behavioral results show that blocking TGM2 provides substantial advantages in neurological function restoration following TBI.

## 4 Discussion

The integrity of the BBB is crucial for maintaining central nervous system (CNS) homeostasis and shielding neural tissue from detrimental systemic factors [30, 31]. TBI induces BBB dysfunction through multilayered pathological mechanisms involving both structural and biochemical alterations [32]. The initial mechanical force directly compromises endothelial tight junction integrity, reducing tight junction proteins, including CLAUDIN‐5, OCCLUDIN, and ZO‐1 [33]. Subsequent inflammatory cascades amplify the barrier breakdown via TNF‐α and IL‐1β, which also upregulate MMP‐9. This protease degrades both basement membrane components and tight junction‐associated proteins [34]. Concurrent oxidative stress exacerbates endothelial dysfunction via reactive oxygen species (ROS)‐induced mitochondrial impairment and apoptotic signaling [35]. Pericyte detachment from microvessels and dysfunction of astrocytic end‐feet compromise the neurovascular unit support system [36]. Pathologically, dysfunctional astrocytic end‐feet secrete vascular endothelial growth factor‐A (VEGF‐A), which elevates vascular permeability [37]. These interrelated mechanisms progressively disrupt the BBB via structural destabilization, proteolytic degradation, redox imbalance, and failure of neuroglial support. In our study, increased TGM2 levels were elevated after TBI, with primary expression observed in mouse brain endothelial cells. As brain endothelial cells form the core structural component of the BBB, TGM2 may serve as a critical regulator of BBB integrity following TBI.

TGM2 displays extraordinary functional multiplicity as a calcium‐reliant multifunctional protease, fundamentally defined by three interlinked biological properties. Its canonical function involves catalyzing calcium‐dependent protein crosslinking through the formation of ε‐(γ‐glutamyl) lysine isopeptide bridges, a post‐translational modification critical for stabilizing both extracellular matrix (ECM) architecture and intracellular protein networks [38]. Beyond this structural role, TGM2 demonstrates pluripotent enzymatic capabilities, including GTPase activity that modulates G protein‐coupled receptor signaling, protein disulfide isomerase (PDI) function in redox regulation, and kinase activity participating in phosphorylation cascades [38]. The enzyme's functional diversity is further amplified by its dynamic subcellular distribution across cytoplasmic compartments, mitochondria, nuclear structures, and extracellular spaces [38, 39]. This positional flexibility is controlled by calcium (Ca^2^)/GTP density fluctuations that trigger reversible structural transitions between its catalytically active “open” mode and dormant “closed” status, permitting context‐specific modulation of its diverse enzymatic tasks in reaction to cellular microenvironmental shifts [40].

Our findings correspond with earlier investigations indicating that TGM2 is involved in vascular remodeling and inflammation, particularly through its role in cell adhesion, ECM remodeling, and protein crosslinking [10]. Existing literature primarily associates TGM2 with fibrosis and neurodegenerative diseases [41], rather than acute neurovascular injury. Nevertheless, our work demonstrated that knockdown of TGM2 restored BBB stability during in vitro experiments. These findings were further validated in vivo experiments through pharmacological inhibition of TGM2 using cystamine. Notably, although cystamine does not directly affect the expression of TGM2, it has been reported that cystamine, as a TGM2 inhibitor, forms a covalent bond with the active site of TGM2, thereby inhibiting its enzymatic activity [42]. Also, one of the key results in our study was the time‐dependent increase in TGM2 expression after TBI, with a maximum at 48 h after injury, which matches the most severe BBB instability [43]. This temporal relationship suggests that TGM2 may be actively involved in the early pathophysiological responses following TBI, potentially serving as an important biomarker for BBB dysfunction. Since earlier research has connected TGM2 to persistent neuroinflammatory states like Alzheimer's disease (AD) and multiple sclerosis (MS) [44, 45], our investigation expands TGM2's function into TBI, emphasizing its potential as a clinical target to safeguard BBB integrity and reduce secondary injury mechanisms. Our inquiry is the initial one to thoroughly examine the function of TGM2 in TBI.

A major contribution of our study is the identification of the TGM2‐IL‐17‐MMP‐9 signaling axis as a critical pathway regulating BBB disruption after TBI. Analysis of the down‐regulated genes from our RNA‐seq results revealed that MMP‐9 expression decreased in the shTGM2 group compared to the shCtrl group after OGD, and KEGG enrichment analysis highlighted the IL‐17 signaling pathway. MMP‐9 is a key enzyme implicated in BBB degradation [46]. MMP‐9 is a well‐established mediator of BBB breakdown in various neurological disorders, including stroke, TBI, and neuroinflammation [47, 48, 49, 50]. MMP‐9 promotes tight junction degradation, increases vascular permeability, and exacerbates neuroinflammation by facilitating leukocyte infiltration into the CNS [51]. Notably, the IL‐17 pathway has been identified as a key upstream regulator of MMP‐9, particularly in neuroinflammatory and autoimmune diseases [52, 53]. Our previous studies have demonstrated that inhibition of MMP‐9 ameliorates BBB disruption [54]. Ni P et al. found that in perioperative neurocognitive disorders (PND), IL‐17A promotes blood–brain barrier disruption and increases MMP‐9 expression [55]. Collectively, our findings, along with previous studies, suggest that knocking down or inhibiting TGM2 may reduce MMP‐9 expression through the IL‐17 signaling pathway, thereby promoting the restoration of BBB integrity following OGD and TBI. These findings align with studies demonstrating that IL‐17 enhances BBB disruption through MMP‐9 activation in conditions such as multiple sclerosis, experimental autoimmune encephalomyelitis (EAE), and ischemic stroke [53, 56, 57]. Nevertheless, the specific function of TGM2 within this nexus remains unexplored. By confirming that TGM2 depletion causes a substantial decrease in MMP‐9 output and a restoration of BBB stability, our investigation establishes an overlooked connection between TGM2 and the IL‐17‐MMP‐9 network. This implies that addressing TGM2 might act as a pioneering therapeutic approach to reduce BBB collapse and neuroinflammation after TBI.

There were several limitations to our research. First, while our findings support a potential role for TGM2 in BBB disruption following TBI, the underlying mechanistic pathways remain to be fully elucidated. Specifically, the involvement of the IL‐17‐MMP‐9 axis in mediating TGM2‐related effects warrants further investigation to clarify the molecular interactions and regulatory mechanisms involved. Second, this study employed the non‐specific TGM2 inhibitor cystamine, which may have introduced off‐target effects. Additionally, the lack of cell‐type‐specific TGM2 knockout models limited our ability to delineate the contribution of endothelial vs. non‐endothelial TGM2. Future studies should employ endothelial cell‐specific *Tgm2* knockout mice, validate the IL‐17 pathway (e.g., IL‐17 neutralizing antibodies or IL‐17 receptor‐deficient models), and extend outcome assessments to chronic phases to evaluate long‐term neurological recovery.

## 5 Conclusion

In summary, we have demonstrated that TGM2 is a pivotal regulator of BBB integrity and neuroinflammation following TBI, with its effects possibly mediated through the IL‐17–MMP‐9 axis. By demonstrating that pharmacological inhibition of TGM2 mitigates BBB disruption, reduces edema, and improves neurological functional outcomes, we highlight its potential as a novel therapeutic target. These findings highlight TGM2 as a critical regulator of BBB integrity after TBI and propose that targeting TGM2 could be a promising therapeutic strategy for preserving BBB function and mitigating neurological deficits following brain injury. Future research should focus on elucidating the precise molecular interactions between TGM2, IL‐17, and MMP‐9, as well as exploring the long‐term effects of TGM2 inhibition on neuroprotection and cognitive recovery.

## Author Contributions

Jieru Yang, Lihan Zhang, and Xiancheng Qiu contributed equally to this work. Jieru Yang designed the study and prepared the manuscript. Jieru Yang, Lihan Zhang, and Xiancheng Qiu performed the experiments. Jieru Yang analyzed the experimental data; Lihan Zhang completed bioinformatics analysis. Jieru Yang, Lihan Zhang, and Xiancheng Qiu jointly completed the crucial experiment for revising the article. Jiyang Wang, Ruicheng Yan, and Jiasen Ye were involved in experiment performance and data collection. Yong Jiang and Jianhua Peng revised the manuscript. Yong Jiang designed the experiments. All the authors reviewed and approved the final version of the manuscript.

## Funding

This work was supported by the grants from the National Natural Science Foundation of China (U24A20689, 82,371,310, 82,271,306), the Sichuan Science and Technology Program (2024ZYD0113, 2023YFH0069, 2023NSFSC0028), the Scientific Research Project of the Sichuan Provincial Health Commission (23LCYJ040), the Luzhou Government‐Southwest Medical University Cooperation Project (2024LZXNYDJ005), and the Southwest Medical University Project (2024ZKZ010).

## Ethics Statement

All animal experiments were approved by the Animal Ethics Committee of the Southwest Medical University Animal Studies Committee (Approval ID: 201903–105). All experimental animal procedures involved in this study were approved by the China Committee for the Care and Use of Laboratory Animals. Every effort was made to minimize the number of animals used and their suffering.

## Conflicts of Interest

The authors declare no conflicts of interest.

## Supporting information


**Figure S1:** TGM2 is one of the core proteins involved in traumatic brain injury (TBI). (A) GO enrichment results of differentially expressed genes (DEGs) at 12 h, 24 h, and 72 h after TBI. (B) Weighted gene co‐expression network analysis (WGCNA): Heatmap of module‐trait correlations from the proteomic data. (C) Intersection of WGCNA modules and differentially expressed genes.

**Figure S2:** Immunohistochemical analysis of edema‐related proteins (AQP4, NKCC1, SUR1, TRPM4) in the ipsilateral cortex of female mice after TBI.

**Figure S3:** Immunohistochemical analysis of edema‐related proteins (AQP4, NKCC1, SUR1, TRPM4) in the ipsilateral cortex of female mice after TBI.

**Figure S4:** TGM2 inhibition modulates the mRNA expression of edema‐related genes and improves cognitive function after TBI.

**Figure S5:** The Y‐maze test after TBI.

**Figure S6:** The Novel Object Recognition (NOR) test after TBI.

**Table S1:** Animal usage.

**Table S2:** Primers of quantitative real‐time PCR.

## Data Availability

The data that support the findings of this study are available from the corresponding author upon reasonable request.
