# Supplementary material for: Inhibition of Transglutaminase 2 Preserves Blood–Brain Barrier Integrity and Improves Neurological Outcomes After Experimental Traumatic Brain Injury in Mice
Source: CNS Neurosci Ther. 2026 Apr 19;32(4):e70887. doi: 10.1002/cns.70887 (PMC13092724; doi:10.1002/cns.70887)
Supplement: Supplementary file 1 — Figure S1: TGM2 is one of the core proteins involved in traumatic brain injury (TBI). (A) GO enrichment results of differentially expressed genes (DEGs) at 12 h, 24 h, and 72 h after TBI. (B) Weighted gene co‐expression network analysis (WGCNA): Heatmap of module‐trait correlations from the proteomic data. (C) Intersection of WGCNA modules and differentially expressed genes. [file CNS-32-e70887-s005.pptx]

## Slide 1
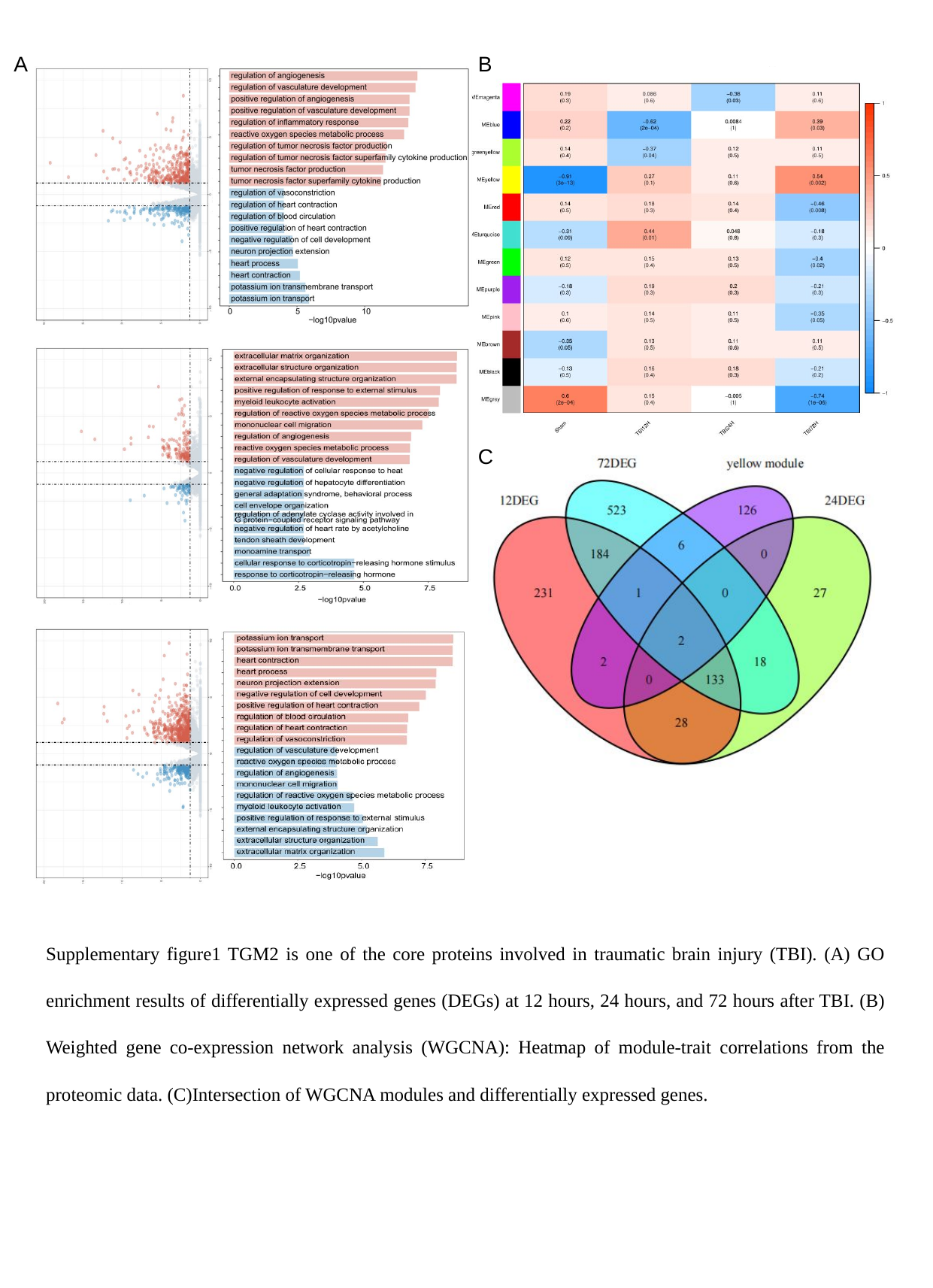

A
B
C
C
Supplementary figure1 TGM2 is one of the core proteins involved in traumatic brain injury (TBI). (A) GO enrichment results of differentially expressed genes (DEGs) at 12 hours, 24 hours, and 72 hours after TBI. (B) Weighted gene co-expression network analysis (WGCNA): Heatmap of module-trait correlations from the proteomic data. (C)Intersection of WGCNA modules and differentially expressed genes.
