# Supplementary figures and images for: Inhibition of Transglutaminase 2 Preserves Blood–Brain Barrier Integrity and Improves Neurological Outcomes After Experimental Traumatic Brain Injury in Mice
Source: CNS Neurosci Ther. 2026 Apr 19;32(4):e70887. doi: 10.1002/cns.70887 (PMC13092724; doi:10.1002/cns.70887)

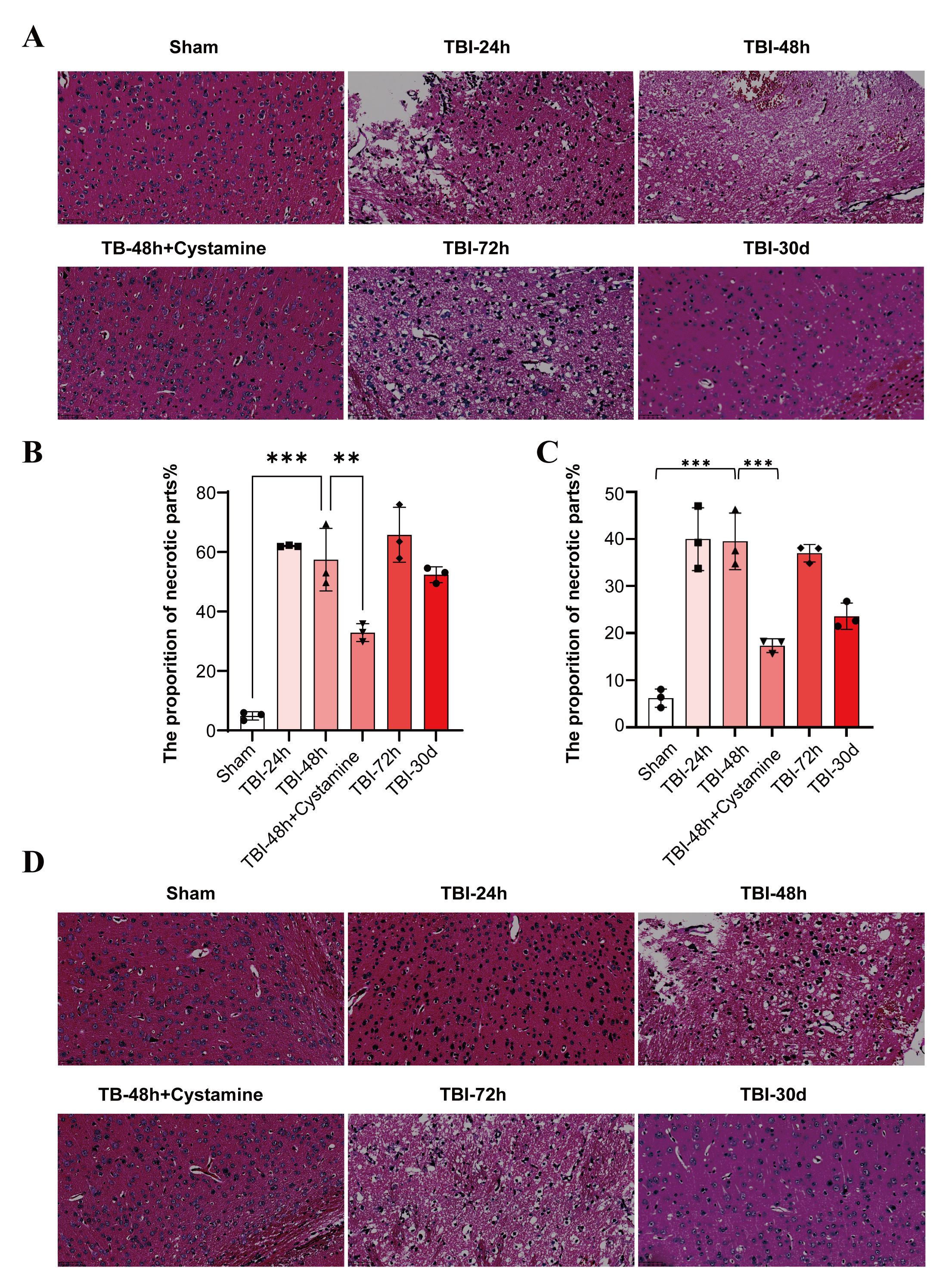

Supplement: Supplementary file 2 — Figure S2: Immunohistochemical analysis of edema‐related proteins (AQP4, NKCC1, SUR1, TRPM4) in the ipsilateral cortex of female mice after TBI. [file CNS-32-e70887-s004.tif]

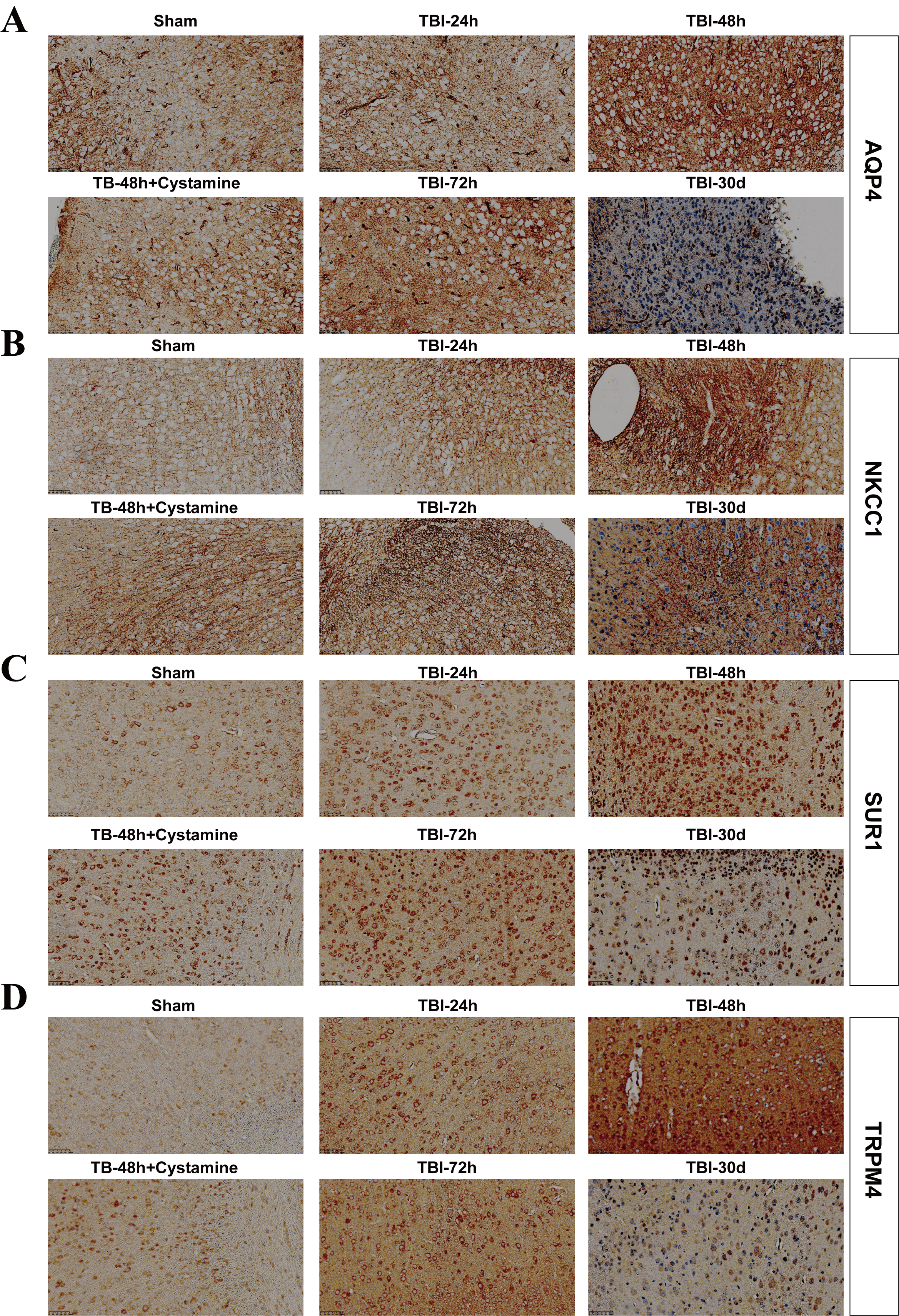

Supplement: Supplementary file 3 — Figure S3: Immunohistochemical analysis of edema‐related proteins (AQP4, NKCC1, SUR1, TRPM4) in the ipsilateral cortex of female mice after TBI. [file CNS-32-e70887-s003.tif]

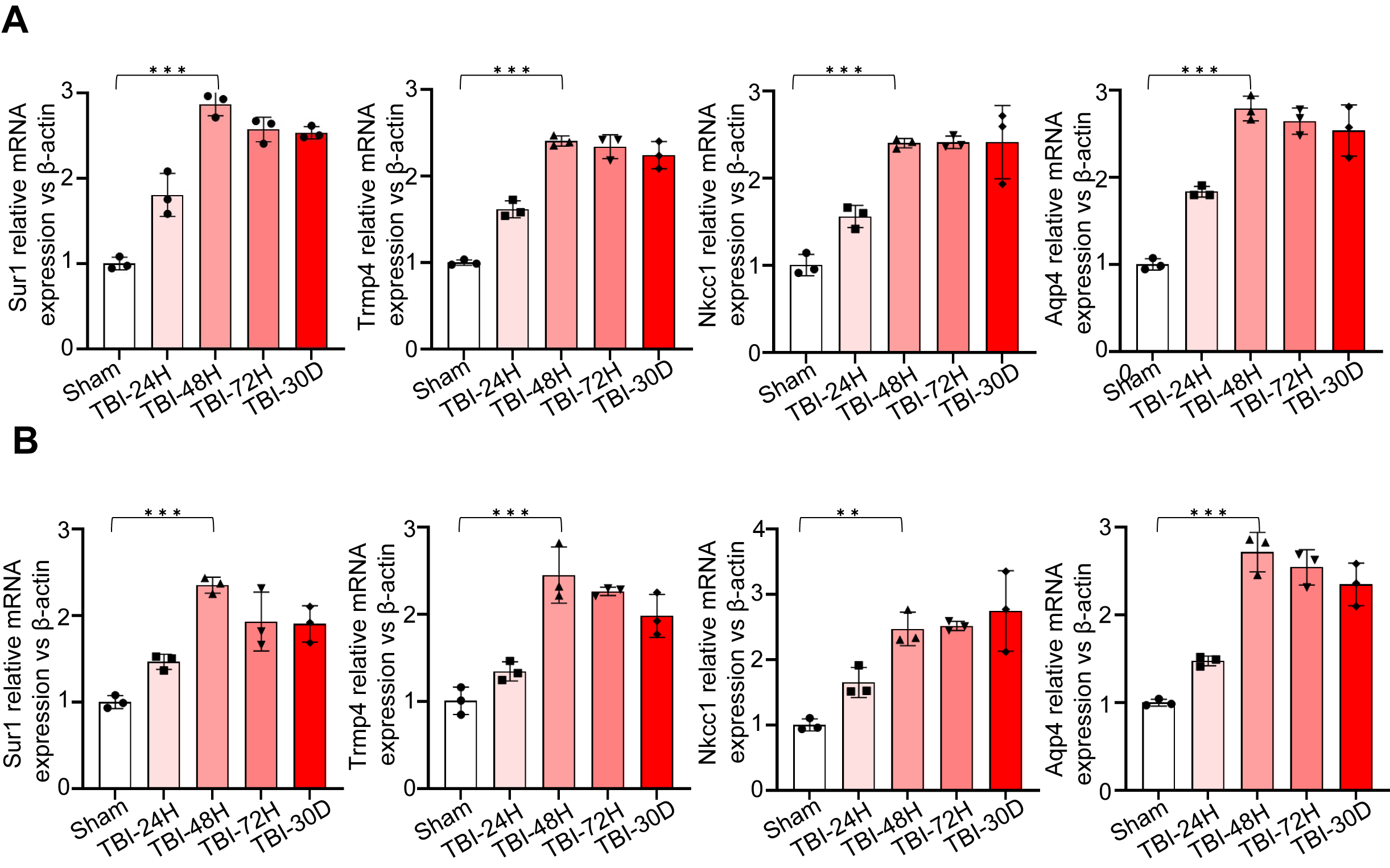

Supplement: Supplementary file 4 — Figure S4: TGM2 inhibition modulates the mRNA expression of edema‐related genes and improves cognitive function after TBI. [file CNS-32-e70887-s002.tif]

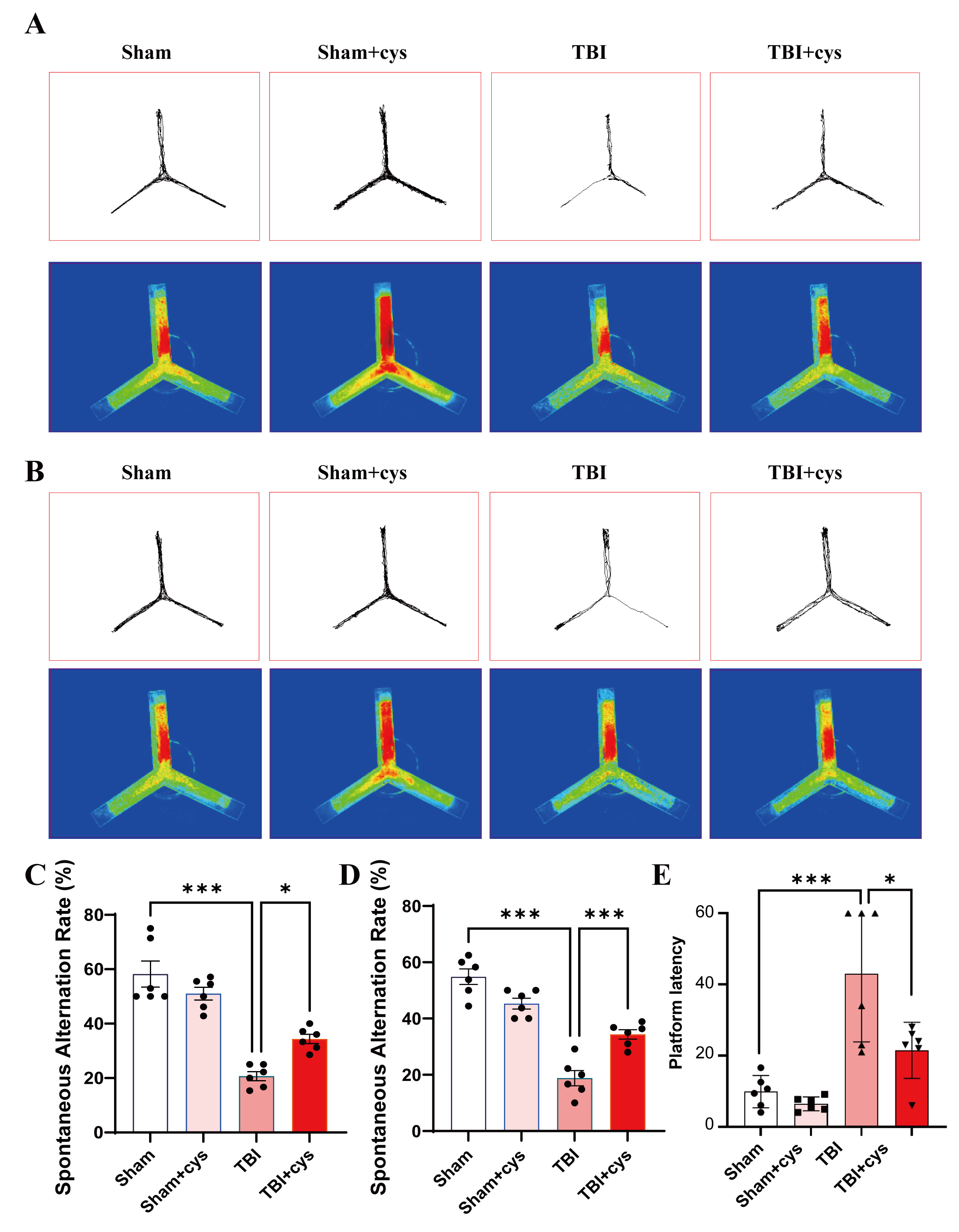

Supplement: Supplementary file 5 — Figure S5: The Y‐maze test after TBI. [file CNS-32-e70887-s006.tif]

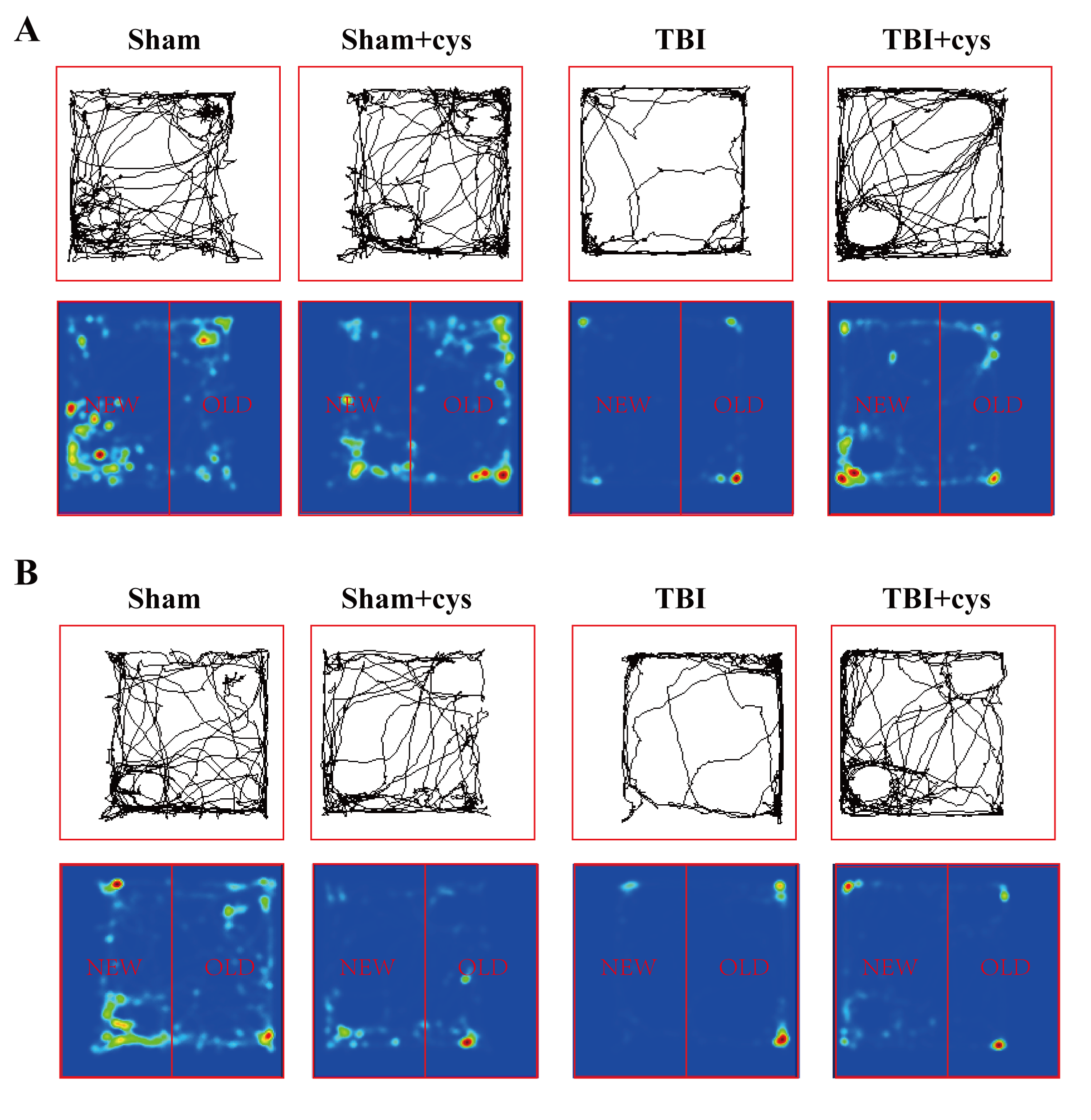

Supplement: Supplementary file 6 — Figure S6: The Novel Object Recognition (NOR) test after TBI. [file CNS-32-e70887-s007.tif]
