# Supplementary material for: Inhibition of Transglutaminase 2 Preserves Blood–Brain Barrier Integrity and Improves Neurological Outcomes After Experimental Traumatic Brain Injury in Mice
Source: CNS Neurosci Ther. 2026 Apr 19;32(4):e70887. doi: 10.1002/cns.70887 (PMC13092724; doi:10.1002/cns.70887)
Supplement: Supplementary file 7 — Table S1: Animal usage. [file CNS-32-e70887-s001.docx]

**Supplementary Table 1 Animal usage**

**This study is reported in accordance with the ARRIVE 2.0 guidelines.**

| **Groups( Male)** | **Mortality rate** |
| --- | --- |
| **Time course** |  |
| Sham | 0 (0/6) |
| TBI(6h, 12h, 24h, 48h, 72h) | 9.09% (3/33) |
|  |  |
| **Exploration of Phenotypes** |  |
| Sham | 0 (0/21) |
| TBI-48h | 8.70% (2/23) |
|  |  |
| **Experiment of intervention** |  |
| Sham+Vehicle | 0 (0/24) |
| Sham+Cystamine | 0 (0/24) |
| TBI(48h)+Vehicle | 11.11% (3/27) |
| TBI(48h)+Cystamine | 7.69% (2/26) |
|  |  |
| **Time course AND 48h of intervention** |  |
| Sham | 0 (18) |
| TBI-24h | 12% (3/25) |
| TBI-48h | 9.52% (2/21) |
| TBI-48h+Cystamine | 8.33% (2/24) |
| TBI-72h | 11.53% (3/26) |
| TBI-30d | 12.5% (3/24) |
|  |  |
| **Total (Male)** |  |
| Sham | 0 (0/93) |
| TBI | 10.31% (23/229) |
|  |  |
| **Groups (Female)** | **Mortality rate** |
| **Time course AND 48h of intervention** |  |
| Sham | 0 (18) |
| TBI-24h | 11.53% (3/26) |
| TBI-48h | 9.09% (2/22) |
| TBI-48h+Cystamine | 8.33% (2/24) |
| TBI-72h | 12% (3/25) |
| TBI-30d | 12% (3/25) |
|  |  |
| **Total (Female)** |  |
| Sham | 0 (0/18) |
| TBI | 10.66% (13/122) |

There experiments were included in this study.

A total of 322 male and 140 female C57BL/6 mice (8-12 weeks of age, 18-25g).
