# Supplementary material for: Inhibition of Transglutaminase 2 Preserves Blood–Brain Barrier Integrity and Improves Neurological Outcomes After Experimental Traumatic Brain Injury in Mice
Source: CNS Neurosci Ther. 2026 Apr 19;32(4):e70887. doi: 10.1002/cns.70887 (PMC13092724; doi:10.1002/cns.70887)
Supplement: Supplementary file 8 — Table S2: Primers of quantitative real‐time PCR. [file CNS-32-e70887-s008.docx]

| **Target** | **Sequence**  **Forward Primer** | **Sequence**  **Reverse Primer** |
| --- | --- | --- |
| **TGM2 primers** | GACAATGTGGAGGAGGGATCT | GACAATGTGGAGGAGGGATCT |
| **Claudin-5 primers** | CTCTGCTGGTTCGCCAACAT | CAGCTCGTACTTCTGCGACA |
| **ZO-1 primers** | CAACATACAGTGACGCTTCACA | CACTATTGACGTTTCCCCACTC |
| **Occludin primers** | TGGCAAGCGATCATACCCAGAG | CTGCCTGAAGTCATCCACACTC |
| **MMP9 primers** | CTGGACAGCCAGACACTAAAG | CTCGCGGCAAGTCTTCAGAG |

**Supplementary Table 2 Primers of quantitative real-time PCR**
